# Association between empirical dietary inflammatory index, odds, and severity of anxiety disorders: A case–control study

**DOI:** 10.1002/fsn3.3573

**Published:** 2023-07-27

**Authors:** Kimia Torabynasab, Hossein Shahinfar, Mohammad Effatpanah, Shima Jazayeri, Leila Azadbakht, Jamileh Abolghasemi, Soulmaz Jamali

**Affiliations:** ^1^ Department of Nutrition, School of Public Health Iran University of Medical Sciences Tehran Iran; ^2^ School of Medicine, Imam Khomeini Hospital Tehran University of Medical Sciences Tehran Iran; ^3^ Department of Community Nutrition, School of Nutritional Sciences and Dietetics Tehran University of Medical Sciences Tehran Iran; ^4^ Department of Biostatistics Iran University of Medical Sciences Tehran Iran; ^5^ Masters in Department of Educational Science Islamic Azad University Science and Research branch Tehran Iran

**Keywords:** anxiety disorders, diet, inflammation, mental disorders, nutrition science

## Abstract

Diet may be a modifiable factor in the prevention of psychiatric disorders by modulating inflammation. In this study, we evaluated the association between empirical dietary inflammatory index (EDII) that is designed to evaluate the inflammatory potential of diets and anxiety disorders (AD) in adults. This case–control study was carried out on 85 patients who were group matched by gender with 170 healthy subjects. Data for dietary intake were assessed by using a 147‐item validated food frequency questionnaire (FFQ). Anthropometric measures were collected using standard methods. EDII score was developed according to participants' dietary intakes of 28 predefined food groups. Multivariate odds ratios (OR) with 95% confidence intervals (CI) were used to investigate the association of empirically derived inflammatory potential of the diet and anxiety disorder. We observed that after adjusting for confounders, individuals in the top category of EDII score were 2.09 fold more likely to have anxiety disorder compared with those in the bottom category (OR: 2.09, 95% CI: 1.01, 4.33). Also, higher EDII contributed to a higher GAD‐7 score (*p* < .001). There was a significant positive linear association between EDII and AD (*β* = 3.64, *p* < .001). After controlling for potential confounders AD had a strong positive correlation with the EDII score (*r* = .61, *p*‐value <.001). In conclusion, in this case–control study, we realized that there is a positive association between the EDII score, odds, and severity of anxiety disorder. Ultimately, the potential role necessitates clarifying this association by conducting large‐population prospective cohort studies.

## Graphical abstract

Diet may be a modifiable factor in the prevention of psychiatric disorders by modulating inflammation. We observed that after adjusting for confounders, individuals in the top category of EDII score were 2.09 fold more likely to have AD compared with those in the bottom category. Also, higher EDII contributed to a higher GAD‐7 score.

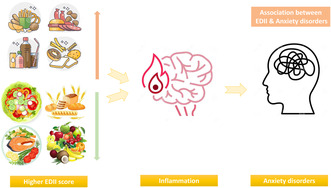

## 1 INTRODUCTION

Anxiety disorders (AD) make up one of the most common groups of psychiatric disorders. They are a group of psychological conditions characterized by significant and uncontrollable feelings of anxiety and fear, affecting a person's social, occupational, and personal functioning significantly. According to the National Comorbidity Survey, one out of four people meets diagnostic criteria for one anxiety disorder (Boland & Verduin, 2021). In Iran, the 12‐month prevalence of anxiety disorders was 15.6%. Males had a prevalence of 12.0%, while females had a prevalence of 19.4% (Hajebi et al., 2018).

Anxiety disorder symptoms indicate exaggerated activation of sympathetic and noradrenergic systems like increased heart rate, chest pain, sweating, etc. Psychiatrists diagnose anxiety disorders based on the Diagnostic and Statistical Manual of Mental Disorders, 5th ed (DSM‐5) criteria which include the type of symptoms, number, and duration of symptoms, as well as other criteria specific to each disorder (Boland & Verduin, 2021).

Paramount supporting evidence indicates that adherence to a high‐quality diet results in protection against psychological health issues which can be attributed to food and dietary components' anti‐ and pro‐inflammatory properties (Wärnberg et al., 2009). Results of previous studies illustrated a positive association between pro‐inflammatory diets, as measured by the dietary inflammatory index (DII), and psychological issues (Adjibade et al., 2017; Bergmans & Malecki, 2017; Phillips et al., 2018). However, some studies reported conflicting findings (Adjibade et al., 2017; Dehghan et al., 2022; Vermeulen et al., 2018). Studies had already established a link between mental issues, including anxiety disorders, and inflammatory dysregulation (Gimeno et al., 2009; Puustinen et al., 2011; Renna et al., 2018; Wium‐Andersen et al., 2013). Evidence demonstrates that elevated pro‐inflammatory cytokine levels result from peripheral cytokine recruitment to the brain and activation of microglia which lead to anxiety disorders (Hu et al., 2022). It should be noted that earlier studies primarily used DII to assess whether the diet is pro‐inflammatory. The construction of DII was based on the scoring of pro‐inflammatory or anti‐inflammatory properties of nutrients (Shivappa et al., 2014). It is of high importance to note that considering the intrinsic interactions of nutrients based on studies focusing on single nutrients is impossible (Cespedes & Hu, 2015). Nutrients are absorbed differently depending on their interaction. Furthermore, variations in the consumption of one food or nutrient can lead to changes in the consumption of others. As a result, diet recommendations should be based on food or food groups rather than nutrients. A holistic view of diet is, therefore, necessary to thoroughly understand how diet is associated with psychological disorders. In this regard, according to the significance of the inflammatory effects of foods, the empirical dietary inflammatory index (EDII) was designed to evaluate the inflammatory potential of diets. The EDII is based on foods and food groups rather than nutrients.

Based on the best information we have, there has been no study investigating the association between anxiety disorders and EDII scores so far. Thus, the purpose of this study was to investigate the relationship between EDII scores in adults with anxiety disorders. Also, the severity of AD was evaluated by Generalized Anxiety Disorder‐7 (GAD‐7) (Johnson et al., 2019).

## 2 SUBJECTS AND METHODS

### 2.1 Participants

This project was performed as a case–control study between 2021 and 2022 on two groups of patients with anxiety disorders (*n* = 85) and healthy people (*n* = 85) in Imam Khomeini Hospital and referral psychology clinics. Subjects were chosen by convenience sampling. A total sample size of 170 individuals was calculated using data from the study and using with 80% power and 95% confidence interval (n=Z1−α2+Z1−β2σ2μ1−μ22, *α* = .05, *β* = .20) (Filippini et al., 2020). Eighty‐five patients were group matched by gender with 170 healthy subjects. To increase the power of the study, however, we continued sampling until the control group had twice as many participants as the case group, and finally, 255 participants enrolled in our study. During the consultation session, the diagnosis was performed and questionnaires were filled out. The patient group included people who have been diagnosed with an anxiety disorder by a psychiatrist using DSM‐5 criteria. The control group included people who came to the psychology clinic for marriage counseling, parenting sessions, or training courses and who did not have anxiety disorders based on DSM‐5 criteria. We considered special criteria for both groups of participants in our study, including not taking antianxiety or antidepression medications within the last 2–3 months, being between the ages of 18 and 60, having a body mass index between 18.5 and 30, and being apparently healthy. We also excluded those who did not answer more than 80% of the questionnaire questions and had a calorie intake of less than 800 or more than 4200 calories per day.

### 2.2 Ethics statement

In accordance with the Declaration of Helsinki, this study was conducted. All participants signed informed consent after receiving all necessary explanations about the project. All participants signed informed consent after receiving all necessary explanations about the project. The study protocol was approved by the Ethics Committee of the Iran University of Medical Science (IR.IUMS.REC.1400.868).

### 2.3 Demographic data collection

Participants' information including sex, age, health status, education, marital status, supplements, medicine intake, and smoking were all collected using self‐administered questionnaires. Our physical activity assessment method was based on a short, valid, and reliable version of the International Physical Activity Questionnaire (IPAQ) (Moghaddam et al., 2012). In the past 7 days, this questionnaire assessed vigorous and moderate activities, walking, and sitting of the subjects. Then, to estimate the total weekly physical activity of each participant, the Metabolic Equivalents (METs) were computed as MET‐min/week. Consequently, subjects were divided into three categories: very low (below 600 MET min/week), low (600–3000 MET‐min/week), moderate, and high (above 3000 MET‐min/week) (Wareham et al., 2003).

### 2.4 Anthropometric data collection

A digital scale (Seca 707; Seca GmbH & Co. KG.) was used to measure weight, and participants were minimally dressed without shoes. Height was measured using a stadiometer (Seca, Germany) in a standing position without shoes to the nearest 0.5 cm. The BMI was calculated by dividing the weight (kg) by the square of the height (m^2^).

### 2.5 Dietary assessment

A validated semiquantitative 147‐item food frequency questionnaire (FFQ) based on Iranian foods was used to assess participants' dietary intake (Mirmiran et al., 2010). Participants were asked two questions for each food item: food consumption frequency (annual, monthly, weekly, or daily in the past year); and approximately how much was consumed each time. The amount and frequency of consumption of all food items were converted to grams per day using “household measures” (Ghafarpour et al., 1999). The total amount of macronutrients and micronutrients consumed was calculated using modified Nutritionist IV software developed for Iranian foods (version 7.0; N‐Squared Computing).

### 2.6 Development of empirical dietary inflammatory index

EDII score was created based on the study conducted by Esmaillzadeh et al. Aiming to construct a score for the overall inflammatory potential of whole diets using food groups, they investigated the association between dietary intakes and systemic inflammation by the evaluation of serum CRP, IL‐6, and TNFα status assessment (Esmaillzadeh et al., 2007). The EDII score incorporates 28 food groups defining the inflammatory potential of the diet. Fruits, fruit juices, whole grains, fish, poultry, cruciferous vegetables, yellow vegetables, green leafy vegetables, other vegetables, legumes, tomatoes, and tea create 12 components of the anti‐inflammatory category, while 16 food groups including processed meat, red meats, eggs, butter, dairy, coffee, potatoes, French fries, refined grains, pizza, snack, mayonnaise, soft drinks, sweet and desserts, hydrogenated fats, and vegetable oils are assigned to the pro‐inflammatory category. Lower scores denote anti‐inflammatory diets, while higher scores indicate pro‐inflammatory diets. Initially, energy‐adjusted amounts for these food groups were calculated using the residual method (Willett et al., 1997), followed by multiplying daily intakes by their factor loadings obtained in Esmaillzadeh et al. studies (Esmaillzadeh et al., 2007). A total EDII score for each participant was then calculated by adding up the scores from each food and food group, then dividing the result by 100 to reduce the magnitude. EDII scores represent the diet's total weight in 28 food groups; with higher scores indicating pro‐inflammatory diets and lower scores indicating anti‐inflammatory diets.

### 2.7 Assessment of anxiety disorder

Expert diagnosis using standard classifying systems like the International Classification of Diseases (ICD) or the American Diagnostic and Statistical Manual (DSM) is the gold standard for diagnosing common mental diseases in clinical and research contexts (Kiely & Butterworth, 2015). In our study, anxiety disorder was diagnosed by a psychiatrist based on DSM‐V criteria. Since the GAD‐7 scale has shown adequate psychometric properties and diagnostic accuracy in the Iranian population, we assessed the severity of anxiety disorder by applying the GAD‐7 questionnaire (Fattah et al., 2021). Total scores of 5, 10, and 15 are considered the cutoff for mild, moderate, and severe anxiety, respectively (Staff, 2008). Each question has a score range from 0 to 3 (0 = not at all, 1 = several days, 2 = more than half the days, and 3 = nearly every day) with a total score between 0 and 21, with higher scores indicating more anxiety.

### 2.8 Statistical analysis

Statistical tests such as Kolmogorov–Smirnov, Shapiro–Wilk, and the Q–Q plot were used to determine the normality of distributions. After that, participants were categorized based on the tertiles of EDII scores. The independent sample t‐test was used to evaluate the differences between the two groups. We used one‐way analysis of variance (ANOVA) for quantitative variables and chi‐square tests for qualitative variables to compare general characteristics among tertiles of the EDII scores. Data were shown as the mean ± SD for continuous variables and percent (%) for categorical ones. Dietary intakes were energy‐adjusted by the residual model (Willett et al., 1997). To compare energy‐adjusted dietary intakes of subjects across tertile of EDII, analysis of covariance (ANCOVA) was applied. We used analysis of covariance (ANCOVA) to compare the GAD‐7 score among tertiles of EDII (adjusted for age, sex, energy intake, marital status, education, medicine use, vitamin supplements use, smoking status, alcohol consumption, physical activity, health status, past medical history, and BMI). Multiple linear regression analysis was used to evaluate the association between AD and EDII score after adjustment for covariates, including age, sex, energy intake, marital status, education, medicine use, vitamin supplements use, smoking status, alcohol consumption, physical activity, health status, past medical history, and BMI. Partial correlation was conducted to assess the relation of AD with test variables including EDII score and its food groups after controlling for potential confounders. We used multinomial logistic regression analysis in different models. First, we controlled for the confounding effect of age, sex, and energy intake. In the second model, we further adjusted for marital status, education, medicine use, vitamin supplements use, smoking status, alcohol consumption, physical activity, health status, and past medical history. In the final model, we additionally adjusted for BMI. All statistical analyses were performed using the Statistical Package for the Social Sciences (SPSS version 25; SPSS Inc.) We considered *p* < .05 as the significance level.

## 3 RESULTS

General characteristics of study participants between cases and controls are presented in Table 1. There were no statistically significant differences in age, weight, height, BMI, sex, marital status, education status, alcohol intake, smoking, dietary supplement use, health status, medication use, and physical activity level between the case and control groups.

**TABLE 1:** General characteristics of patients with anxiety disorders and controls.

	Control (170)	Case (85)	*p*‐value^a^
Age (year)	33.6 ± 11.2	35.5 ± 12.7	.22
Weight (kg)	69.3 ± 12.9	68.1 ± 13.3	.48
Height (cm)	166 ± 12.9	165 ± 8.85	.68
BMI (kg/m^2^)	25.7 ± 12.9	24.7 ± 4.25	.46
Gender, *n* (%)			
Male	49 (64.5)	27 (35.5)	.62
Female	121 (67.6)	58 (32.4)	
Marital status, *n* (%)			
Married	80 (63.5)	46 (36.5)	.28
Single	90 (69.8)	39 (30.2)	
Education status, *n* (%)			
Undergraduate	133 (68.9)	60 (31.3)	.37
Diploma	6 (54.5)	5 (45.5)	
College education	31 (60.8)	20 (39.2)	
Alcohol, *n* (%)			
Yes	14 (70.0)	6 (30.0)	.74
No	156 (66.4)	79 (33.6)	
Smoke, *n* (%)			
Yes	23 (57.5)	17 (42.5)	.18
No	147 (68.4)	68 (31.6)	
Health status *n* (%)			
Healthy	156 (66.1)	80 (33.9)	.50
Nonhealthy	14 (73.7)	5 (26.30)	
Past medical history *n* (%)			
Healthy	160 (68.1)	75 (31.9)	.10
Nonhealthy	10 (50.0)	10 (50.0)	
Dietary supplement use, *n* (%)			
Yes	105 (70.9)	43 (29.1)	.08
No	65 (60.70)	42 (39.3)	
Medication use, *n* (%)			
Yes	21 (77.8)	6 (22.2)	.19
No	149 (65.4)	79 (34.6)	
Activity level, *n* (%)			
Low	67 (61.5)	42 (38.5)	.28
Moderate	64 (71.9)	25 (28.1)	
High	39 (68.4)	18 (31.6)	

*Note*: Values are based on mean ± SD or reported percentage. Chi‐squared test for categorical variables and Student's *t* test for continuous variables have been used.

Abbreviations: BMI, body mass index; cm, centimeter; Kg/m^2^, kilogram/meter; *n*, number.

^a^ *p*‐value <.05 was considered significant.

General characteristics of study participants according to tertiles of EDII are presented in Table 2. Compared to those in the lowest tertile, subjects in the top tertile were older, had lower heights, and had higher BMI. Distribution of past medical history, medication use, marital status, and education status was different across tertiles of EDII score. There was no significant difference in the distribution of the status of education, smoking, and other medical histories of participants across tertiles of EDII score. There were no significant differences in other characteristics across tertiles of EDII, as well.

**TABLE 2:** General characteristics of the participants in the study across tertiles of EDII.

*n*	EDII			*p*‐Value
	T1 85	T2 85	T3 85	
EDII score range	(1.20, 7.20)	(7.21, 10.2)	(10.21, 30.9)	
Age (year)	31.7 ± 10.8	31.2 ± 9.57	39.7 ± 12.7	<.001
Weight (Kg)	68.1 ± 13.7	68.7 ± 12.7	69.7 ± 12.7	.719
Height (m)	168 ± 9.95	168 ± 9.33	162 ± 14.3	.001
BMI (kg/m^2^)	24.0 ± 4.4	24.2 ± 3.82	27.9 ± 17.6	.003
Sex, *n* (%)				
Male	23 (30.3)	31 (40.8)	22 (28.9)	.255
Female	62 (34.6)	54 (30.2)	63 (35.2)	
Current health status *n* (%)				
Healthy	79 (33.5)	83 (35.2)	74 (31.4)	.31
Nonhealthy	62 (31.6)	2 (10.5)	11 (57.9)	
Past medical history *n* (%)				
Healthy	81 (34.5)	82 (34.9)	72 (30.6)	.007
Nonhealthy	4 (20.0)	3 (15.0)	13 (65.0)	
Medication *n* (%)				
Yes	78 (34.2)	82 (36.0)	68 (29.8)	.002
No	7 (25.0)	3 (11.1)	17 (63.0)	
Supplement *n* (%)				
Yes	32 (29.9)	43 (40.2)	32 (29.9)	.143
No	53 (35.8)	42 (28.4)	53 (35.8)	
Marital status *n* (%)				
Single	54 (41.9)	53 (41.4)	22 (17.1)	<.001
Married	31 (24.6)	32 (25.4)	63 (50)	
Education *n* (%)				
Undergraduate	2 (18.2)	2 (18.2)	7 (63.6)	.042
Diploma	15 (29.4)	13 (25.5)	23 (45.1)	
College education	68 (35.2)	70 (36.3)	55 (28.5)	
Smoking, *n* (%)				
Yes	10 (25.0)	17 (42.5)	13 (32.5)	.334
No	75 (34.9)	68 (31.6)	72 (33.5)	
Alcohol, *n* (%)				
Yes	5 (25.0)	6 (30.0)	9 (45,0)	.494
No	80 (34.0)	79 (33.6)	76 (32.3)	
Physical activity, *n* (%)				
Low	38 (34.9)	31 (28.4)	40 (36.7)	.151
Moderate	34 (38.2)	28 (31.5)	27 (30.3)	
High	13 (22.8)	26 (45.6)	18 (31.6)	

*Note*: *p*‐value <.05 was considered significant. Values are based on mean ± SD or reported percentage. One‐way analysis of variance (ANOVA) for quantitative data and Chi‐squared test for qualitative data have been used. Subjects in the first tertile of EDII had EDII score between 1.20 and 7.20; second tertile: between 7.21 and 10.20; third tertile: between 10.21 and 30.9.

Abbreviations: BMI, body mass index; cm, centimeter; EDII, empirically dietary inflammatory index; kg, kilogram; m, meter; *n*, numbers.

Food groups according to the tertiles of the EDII are indicated in Table 3. Individuals in the third tertile of EDII had a higher intake of refined grains (*p* < .001), legumes (*p* = .008), red meat (*p* < .001), other vegetables (*p* = .002), tomatoes (*p* < .001), poultry (*p* = .005), vegetable oils (*p* = .001), soft drinks (*p* = .03), tea (*p* < .001), potato (*p* = .005), dairy (*p* < .001), fruits (*p* < .001) as well as lower intake of mayonnaise (*p* = .01) and pizza (*p* < .001). The intake of other food groups was not significantly different across tertiles of EDII.

**TABLE 3:** Dietary intakes of the study participants according to Tertiles of EDII.

Tertiles of EDII				
	T1	T2	T3	*p*‐Value
EDII score range	(1.20, 7.20)	(7.21, 10.2)	(10.21, 30.9)	
Subjects, *n*	85	85	85	
Refined grains, g/d	132 ± 115	218 ± 165	312 ± 227	<.001
Whole grains, g/d	40.9 ± 103	48.3 ± 50.0	56.2 ± 57.7	.40
Legumes, g/d	2.37 ± 6.62	11.3 ± 21.0	16.2 ± 45.5	.008
Red meat, g/d	46.6 ± 52.3	95.1 ± 101	80.7 ± 66.9	<.001
Processed meat, g/d	21.2 ± 41.2	17.9 ± 34.9	21.5 ± 48.3	.83
Green leafy vegetables, g/d	22.4 ± 30.3	25.3 ± 16.7	25.6 ± 10.2	.55
Cruciferous vegetables, g/d	10.6 ± 34.8	7.50 ± 17.4	8.29 ± 11.1	.67
Yellow vegetables, g/d	14.2 ± 35.5	15.8 ± 34.2	7.39 ± 13.5	.14
Other vegetables, g/d	33.4 ± 50.1	51.0 ± 47.0	66.0 ± 77.9	.002
Tomatoes, g/d	24.5 ± 47.4	46.1 ± 53.6	121 ± 272	<.001
Poultry, g/d	11.4 ± 55.7	19.8 ± 37.0	35.1 ± 48.8	.005
Vegetable Oils, g/d	1.02 ± 10.7	3.54 ± 7.39	6.62 ± 10.5	.001
Soft Drinks, g/d	86.1 ± 255	140 ± 280	208 ± 367	.03
Sweets and dessert, g/d	227 ± 203	188 ± 148	144 ± 196	.06
Mayonnaise, g/d	2.71 ± 4.90	1.40 ± 3.36	1.19 ± 2.18	.01
Tea, g/d	106 ± 150	211 ± 196	626 ± 884	<.001
Coffee, g/d	18.8 ± 109	52.9 ± 235	12.5 ± 80.7	.19
Snacks, g/d	18.0 ± 36.9	10.6 ± 24.4	9.78 ± 12.3	.08
Butter, g/d	3.89 ± 12.8	2.61 ± 6.27	5.54 ± 7.47	.12
French fries, g/d	4.69 ± 9.51	3.73 ± 5.89	4.82 ± 6.28	.58
Potato, g/d	5.86 ± 23.4	15.9 ± 29.0	6.46 ± 9.40	.005
Dairy, g/d	191 ± 173	270 ± 145	348 ± 250	<.001
Fruits, g/d	89.7 ± 126	263 ± 185	459 ± 299	<.001
Fruits juices, g/d	54.4 ± 118	34.8 ± 69.8	53.6 ± 101	.34
Fish, g/d	5.60 ± 25.5	5.56 ± 17.9	4.95 ± 17.1	.97
Egg, g/d	9.27 ± 25.5	21.6 ± 25.4	24.4 ± 20.6	<.001
Hydrogenated fats, g/d	6.84 ± 10.4	5.70 ± 10.4	3.71 ± 16.4	.27
Pizza, g/d	93.0 ± 140	55.4 ± 119	21.9 ± 44.7	<.001

*Note*: All values were adjusted for energy intake using analysis of covariance (ANCOVA). Values are based on mean ± SD. *p*‐value <.05 was considered significant.

Abbreviations: EDII, empirical dietary inflammatory index; g/d, gram per day.

The multivariate‐adjusted means for the GAD‐7 score according to the tertiles of EDII, which represents the severity of the anxiety disorder in individuals suffering from it, is shown in Table 4. In the crude model, we observed that higher EDII was associated with a higher GAD‐7 score (*p* < .001). After controlling for covariates, these results remained significant (*p* < .001), as well.

**TABLE 4:** Multivariate‐adjusted means for GAD‐7 score across tertiles of EDII.

	Tertiles of EDII						
	T1	T2	T3	P_1_	P_2_	P_3_	P_4_
Tertiles of EDII (range)	(1.20, 7.20)	(7.21, 10.2)	(10.21, 30.9)				
*n*	23	26	36				
GAD‐7 score	3.48 ± 2.04	5.04 ± 1.80	13.9 ± 3.91	<0.001	<0.001	<0.001	<0.001

*Note*: *p*‐value <.05 was considered significant. Values are based on mean ± SD. *p*‐values derived from analysis of covariance (ANCOVA). P_1_: crude model. P_2_: adjusted for age, sex, and energy intake. P_3_: additionally, adjusted for marital status, education, medicine use, vitamin supplements use, smoking status, alcohol consumption, physical activity, health status, and past medical history. P_4_: additionally, adjusted for BMI.

Abbreviations: EDII, empirical dietary inflammatory index; GAD‐7, Generalized anxiety disorder‐7.

Multivariate‐adjusted odds ratios and 95% confidence intervals for AD across tertiles of EDII are presented in Table 5. In the crude model, those who were in the third tertile of EDII compared to the first tertile were more likely to have AD (OR = 1.98; 95% CI:1.04, 3.76). Such a significant association was also seen after taking age, sex, energy intake, marital status, education, medicine use, vitamin supplements use, smoking status, alcohol consumption, physical activity, health status, and past medical history into account. Additional adjustments for BMI revealed a significant positive association between EDII and odds of AD; such that individuals in the top category of EDII score were 2.09 fold more likely to have AD compared with those in the bottom category (OR: 2.09, 95% CI: 1.01, 4.33).

**TABLE 5:** Odds ratios (ORs) and 95% confidence intervals (95% CIs) for anxiety disorders according to tertiles of EDII score.

	Tertiles of EDII			*p* trend
	T1	T2	T3	
Mean EDII score	4.21	4.44	12.46	
	OR	OR (95% CI)	OR (95% CI)	
Crude	1	1.18 (0.61, 2.39)	1.98 (1.04, 3.76)	0.37
Model I	1	1.18 (0.06, 2.30)	1.88 (0.96, 3.69)	0.63
Model II	1	1.13 (0.56, 2.27)	2.01 (0.97, 4.16)	0.05
Model III	1	1.15 (0.57, 2.31)	2.09 (1.01, 4.33)	0.04

*Note*: Data are OR (95% CI). Model I: adjusted for age, sex, and energy intake. Model II: additionally, adjusted for marital status, education, medicine use, vitamin supplements use, smoking status, alcohol consumption, physical activity, health status, and past medical history. Model III: additionally, adjusted for BMI.

Abbreviation: EDII, Empirical dietary inflammatory index.

We assessed the relationship between EDII with potential predictor variables of AD and demographic variables in Table 6. There was a significant positive linear association between EDII and AD (*β* = 4.12, *p* < .001). Moreover, after controlling for covariates, the result remained unchanged (*β* = 3.64, *p* < .001).

**TABLE 6:** Association between GAD‐7 score and EDII.

	EDII			
	*β* ± SE	95% CI	a*R* ^2^	*p*‐Value
GAD‐7 score				
Crude	4.12 ± 0.25	3.61, 4.63	.50	<.001
Model I	3.73 ± 0.25	3.23, 4.23	.55	<.001
Model II	3.63 ± 0.26	3.11, 4.15	.56	<.001
Model III	3.64 ± 0.26	3.12, 4.16	.55	<.001

*Note*: *p*‐value <.05 was considered significant.*p*‐value obtained from linear regression. Model I: adjusted for age, sex, and energy intake. Model II: additionally, adjusted for marital status, education, medicine use, vitamin supplements use, smoking status, alcohol consumption, physical activity, health status, and past medical history. Model III: additionally, adjusted for BMI.

Abbreviations: a*R* ^2^, adjusted R square; CI, confidence interval; EDII, Empirical dietary inflammatory index; SE, standard error; *β*, unstandardized coefficients.

The partial correlation between AD and EDII and its food groups is provided in Table 7. After controlling for potential confounders, AD had a strong positive correlation with EDII score (*r* = .61, *p*‐value <.001), while the positive correlation between AD and refined grains (*r* = 0.31, *p*‐value <.001), fruits (*r* = .40, *p*‐value <.001) was medium. on the other hand, the positive correlation between AD and tomatoes (*r* = .27, *p*‐value <.001), poultry (*r* = .18, *p*‐value = .004), vegetable oils (*r* = .14, *p*‐value = .02), tea (*r* = .27, *p*‐value <.001), dairy (*r* = .16, *p*‐value = .01) was small. In addition, there was a negative small correlation between AD and pizza (*r* = −.25, *p*‐value <.001).

**TABLE 7:** Partial correlation of EDII and its food groups with anxiety disorder score.

	AD	
	Pearson partial correlation **(** *r*)	*p*‐Value
EDII score	.61	<.001
Refined grains, g/day	.31	<.001
Whole grains, g/day	.07	.26
Legumes, g/day	.07	.78
Red meat, g/day	.04	.51
Processed meat, g/day	.02	.68
Green leafy vegetables, g/day	−.04	.44
Cruciferous vegetables, g/day	−.01	.78
Yellow vegetables, g/day	−.06	.28
Other vegetables, g/day	.05	.43
Tomatoes, g/day	.27	<.001
Poultry, g/day	.18	.004
Vegetable Oils, g/day	.14	.02
Soft Drinks, g/day	.12	.05
Sweets and dessert, g/day	−.1	.12
Mayonnaise, g/day	−.11	.08
Tea, g/day	.27	<.001
Coffee, g/day	−.04	.50
Snacks, g/day	−.07	.26
Butter, g/day	.04	.49
French fries, g/day	−.03	.63
Potato, g/day	−.07	.21
Dairy, g/day	.16	.01
Fruits, g/day	.40	<.001
Fruits juices, g/day	.03	.62
Fish, g/day	.00	.98
Egg, g/day	.12	.05
Hydrogenated fats, g/day	−.10	.10
Pizza, g/day	−.25	<.001

*Note*: Data are pearson partial correlation coefficients.

Abbreviation: EDII, empirical dietary inflammatory index.

## 4 DISCUSSION

Our case–control study demonstrates that after adjustment for major covariates, the pro‐inflammatory diet is associated with increased anxiety disorders in the apparently healthy adult population. In addition, the severity of anxiety disorders, assessed by GAD‐7, and EDII were positively associated. We also realized that individuals in the third tertile of EDII had a higher intake of some particular food groups creating EDII scores such as refined grains, legumes, red meat, other vegetables, tomatoes, poultry, vegetable oils, soft drinks, tea, potatoes, dairy, and fruits, while had a lower intake of mayonnaise and pizza. Individuals in the top category of EDII score were 2.09 fold more likely to have AD compared with those in the bottom category. To the best of our knowledge, this is the first study examining the association between EDII, odds, and severity of anxiety disorders.

In the present study, we found a positive relationship between the odds of anxiety disorders and EDII scores. Our results were in line with other studies investigating the association between pro‐inflammatory diet and psychiatric disorders. According to one of the studies of this kind, Belliveu et al. have shown that a pro‐inflammatory EDII score is associated with increased depressive symptoms (Belliveau et al., 2022). Similarly, Burrows et al. found that individuals with major depressive disorders (MDD) with decreased appetite had higher DII scores than those with major depressive disorders with increased appetite and healthy comparisons. They found that anxiety severity was linked to higher DII only in patients with MDD and decreased appetite (Burrows et al., 2020). Our findings were also consistent with an earlier cross‐sectional study conducted by Moludi et al., investigating various aspects of diets with depression was shown that high DII scores are associated with an increased risk of depression as well (Moludi et al., 2020). The cross‐sectional and longitudinal investigation performed on a community‐based cohort by Shakya et al., compared the highest and the lower quartile of EDII, depression risk was increased in the highest one. In the mentioned study, the association was slightly stronger in males than females when stratified by sex. Possibly, this difference is due to differences in food choices based on sex (Shakya et al., 2021). Similar to the current study, Salari Moghadam et al., revealed that higher empirically derived food‐based DII (FDII) was associated with a higher risk of being in the top tertile of psychological disorders including depression, anxiety, and psychological distress. Conducting gender‐stratified analysis, this significant association was observed in women only (Salari‐Moghaddam et al., 2021). Also, our results were commensurate with a cross‐sectional study performed by Haghighatdoost et al., showing associations between lower DII and lower risk of mental health disorders. In this study, after gender‐stratified analysis, the associations were attenuated in men, while remaining significant in women (Haghighatdoost et al., 2019). A meta‐analysis of 11 studies containing 101,950 participants conducted in 2018 demonstrates that comparing those on anti‐inflammatory diets, people eating a pro‐inflammatory diet are 1.4 times more likely to be diagnosed with depression or show depressive symptoms (Tolkien et al., 2019).

Contrasting our results, in a recent cross‐sectional study carried out by Dehghan et al., a nonsignificant positive relationship was seen between DII and mental health profile score including depression, anxiety, and stress in Iranian female adolescents (Dehghan et al., 2022). The results inconsistency may be due to differences in dietary and psychological assessment tools. We used FFQ in our study while in the mentioned study, the 3‐day food record was taken into account. Also, the DASS questionnaire, which is a Depression Anxiety Stress Scale, is not particularly developed for anxiety disorders, while GAD‐7 used in our study is specifically developed to evaluate anxiety disorders (Beard & Björgvinsson, 2014). It is worth noting that in the mentioned study, the association between DII and psychological issues was investigated, while we calculated EDII which considers intrinsic interactions of nutrients. In another large prospective study conducted by Adjibade et al. except in a specific subgroup, no association was found between a pro‐inflammatory diet, indexed by DII, and depressive symptoms (Adjibade et al., 2017). Also, in a cohort study conducted by Vermeulen et al., no longitudinal association between adherence to an inflammatory dietary pattern and higher depressive symptoms was found (Vermeulen et al., 2018). These contrasting results might be attributed to diversities in dietary and psychological assessment tools, and the different psychological outcomes, (CES‐D) scale used to assess depressive symptoms, as well. Additionally, Philips et al., reported that a 70% higher risk of depressive symptoms, a 60% higher risk of anxiety, and a 38% lower risk of reporting good well‐being was demonstrated by participants with the highest EDII; however, these findings no longer persisted when adjustment for depression history and antidepressant medication use was made (Phillips et al., 2018). In their study, no consumption of antianxiety and depressive drugs was considered as an inclusion criterion, and thus their possible effect on the results was neutralized. In another study conducted by Wirth et al., analyzing data from the United States National Health and Nutrition Examination Survey (NHANES), no significant association between depressive symptoms and DII was found (Wirth et al., 2017). Similarly, in another study using NHANES data performed by Bergman et al., it is shown that comparing individuals in the third quintile with the first one, there is no significant association between frequent anxiety and DII (Bergmans & Malecki, 2017). On the other hand, results of a longitudinal study performed by Akbaralay et al., in 2016, a sex‐specific association between DII and recurrent depressive symptoms was shown in a way that over the 5‐year follow‐up, the risk of the depressive symptoms development was higher in women compared to men (Akbaraly et al., 2016). Various exposure (DII) and outcome (depression) considerations might be the reason for opposing results.

A recently published review article by Li et al. describes how a pro‐inflammatory diet might contribute to the etiology of psychological disorders. Diets with pro‐inflammatory properties, presented by high DII and EDII, contribute to chronic activation of immune cells, polymorphonuclear leukocytes in particular, leading to excessive production and accumulation of reactive oxygen species. Additionally, it is hypothesized that the neurons of the brain are more susceptible to oxidative stress due to higher oxygen requirements and a weak antioxidant defense. ROS accumulation triggers inflammatory factors like NLRP3 (NOD‐, LRR‐, and pyrin domain‐containing protein 3). This cytoplasmic protein complex activates caspase‐1 which contributes to promotions in pro‐inflammatory cytokines production like IL1b. Furthermore, ROS can regulate inflammation by activating the transcription factors like nuclear factor‐kappa B (NF‐kb) and activator protein‐1 (AP‐1) which results in the overproduction of pro‐inflammatory cytokines. These cytokines play destructive roles by either indoleamine 2.3‐dioxygenase (IDO) stimulation leading to neurotoxic quinolinic acid production or affecting on hypothalamic–pituitary–adrenal (HPA) axis. The latter gives rise to diminished neuronal plasticity and hippocampal size as well as compromised neurochemical function (Li et al., 2021).

Specific nutrients in the diet are involved in anti‐inflammatory activities (Calder, 2017; Cannell et al., 2014; Hosseini et al., 2021; Moosavian et al., 2020). For instance, vitamin D reduces inflammation by manipulating both innate and adaptive immune systems. In other words, the general impact is a transition from more inflammatory T‐helper responses to less inflammatory ones leading to a sharp decrease in pro‐inflammatory markers production like tumor necrosis factor α (TNF‐ α), interferon‐gamma (IFN‐ γ), interleukin (IL)‐2, IL‐12, IL‐17, and IL‐21 while increasing the production of anti‐inflammatory cytokines such as IL‐10 (Aranow, 2011; Guillot et al., 2010). Other important anti‐inflammatory agents in diet are omega‐3 fatty acids including eicosapentaenoic acid (EPA) and docosahexaenoic acid (DHA). The mechanisms elucidating the anti‐inflammatory effects of EPA and DHA encompass modified cellular membrane phospholipid fatty acid constitution, disturbance of lipid rafts, impediment of the initiation of the pro‐inflammatory transcription factor nuclear factor κB, thereby mitigating the manifestation of inflammatory genes and stimulation of the anti‐inflammatory transcription factor peroxisome proliferator‐activated receptor γ (Calder, 2017).

This study has remarkable strengths worth considering. This is the first study evaluating the relation between anxiety disorders, not anxiety symptoms, and EDII. In addition, participants were classified into case and control groups based on psychiatrist diagnosis using DSM‐5 criteria. Applying a validated food frequency questionnaire (FFQ) to assess dietary intakes, and GAD‐7 for anxiety severity evaluation provided us with precise information as well.

Despite the strength, there are limitations that should be taken into account. Firstly, this was a case–control study and the temporal relationship is not clarified. Secondly, we may have encountered a measurement error when evaluating food intake because of the FFQ's dependence on memory. Getting help from experts and increasing reviews helped us narrow down these issues. Thirdly, although some potential confounders were adjusted for, we could not entirely intercept confounding from unmeasured variables. Ultimately, we should declare that the small sample size is one of the important limitations of our study.

## 5 CONCLUSION

In conclusion, in this case–control study, we realized that there is a positive association between the EDII score and the odds of anxiety disorder. Also, there is a positive relationship between the severity of AD assessed by GAD‐7 and EDII score. Additionally, a positive correlation between food groups creating EDII scores and AD was found as well. Ultimately, the potential role of diet as a modifiable factor necessitates clarifying the association between food inflammatory properties and mental health issues by conducting large‐population prospective cohort studies.

## AUTHOR CONTRIBUTIONS

**Kimia Torabynasab:** Conceptualization (equal); data curation (equal); writing – original draft (equal). **Hossein Shahinfar:** Formal analysis (equal); writing – review and editing (equal). **Mohammad Effatpanah:** Data curation (equal); resources (equal); supervision (equal). **Shima Jazayeri:** Resources (equal); supervision (equal); writing – review and editing (equal). **Leila Azadbakht:** Methodology (equal). **Jamileh Abolghasemi:** Formal analysis (equal). **Soulmaz Jamali:** Methodology (equal).

## FUNDING INFORMATION

This work is supported by the Vice‐Chancellor for Research, Iran University of Medical Sciences [research no. 1400‐3‐2‐22122].

## CONFLICT OF INTEREST STATEMENT

None.

## Data Availability

The datasets generated or analyzed during the current study are not publicly available but are available from the corresponding author on reasonable request.
